# The carbapenem resistance gene *bla*_OXA-23_ is disseminated by a conjugative plasmid containing the novel transposon Tn*6681* in *Acinetobacter johnsonii* M19

**DOI:** 10.1186/s13756-020-00832-4

**Published:** 2020-11-09

**Authors:** Gongli Zong, Chuanqing Zhong, Jiafang Fu, Yu Zhang, Peipei Zhang, Wenchi Zhang, Yan Xu, Guangxiang Cao, Rongzhen Zhang

**Affiliations:** 1 Key Laboratory of Industrial Biotechnology of Ministry of Education and School of Biotechnology, Jiangnan University, Wuxi, 214122 China; 2 Department of Epidemiology, The First Affiliated Hospital of Shandong First Medical University, Jinan, 250062 China; 3 Shandong Medicinal Biotechnology Center, Shandong First Medical University and Shandong Academy of Medical Sciences, Jinan, 250062 China; 4 School of Municipal and Environmental Engineering, Shandong Jianzhu University, Jinan, 250101 China; 5 Key Lab for Biotech-Drugs of National Health Commission, Jinan, 250062 China; 6 Solomon H. Snyder Department of Neuroscience, Johns Hopkins University School of Medicine, Baltimore, MD 21205 USA

**Keywords:** *Acinetobacter johnsonii*, Carbapenem resistance, Conjugative plasmid, Novel transposon Tn*6681*, *bla*_OXA-23_

## Abstract

**Background:**

Carbapenem resistant *Acinetobacter* species have caused great difficulties in clinical therapy in the worldwide. Here we describe an *Acinetobacter johnsonii* M19 with a novel *bla*_OXA-23_ containing transposon Tn*6681* on the conjugative plasmid pFM-M19 and the ability to transferand carbapenem resistance.

**Methods:**

*A. johnsonii* M19 was isolated under selection with 8 mg/L meropenem from hospital sewage, and the minimum inhibitory concentrations (MICs) for the representative carbapenems imipenem, meropenem and ertapenem were determined. The genome of *A. johnsonii* M19 was sequenced by PacBio RS II and Illumina HiSeq 4000 platforms. A homologous model of OXA-23 was generated, and molecular docking models with imipenem, meropenem and ertapenem were constructed by Discovery Studio 2.0. Type IV secretion system and conjugation elements were identified by the Pathosystems Resource Integration Center (PATRIC) server and the oriTfinder. Mating experiments were performed to evaluate transfer of OXA-23 to *Escherichia coli* 25DN.

**Results:**

MICs of *A. johnsonii* M19 for imipenem, meropenem and ertapenem were 128 mg/L, 48 mg/L and 24 mg/L, respectively. Genome sequencing identified plasmid pFM-M19, which harbours the carbapenem resistance gene *bla*_OXA-23_ within the novel transposon Tn*6681*. Molecular docking analysis indicated that the elongated hydrophobic tunnel of OXA-23 provides a hydrophobic environment and that Lys-216, Thr-217, Met-221 and Arg-259 were the conserved amino acids bound to imipenem, meropenem and ertapenem. Furthermore, pFM-M19 could transfer *bla*_OXA-23_ to *E. coli* 25DN by conjugation, resulting in carbapenem-resistant transconjugants.

**Conclusions:**

Our investigation showed that *A. johnsonii* M19 is a source and disseminator of *bla*_OXA-23_ and carbapenem resistance. The ability to transfer *bla*_OXA-23_ to other species by the conjugative plasmid pFM-M19 raises the risk of spread of carbapenem resistance.

**Graphic abstract:**

The carbapenem resistance gene *bla*_OXA-23_ is disseminated by a conjugative plasmid containing the novel transposon Tn*6681* in *Acinetobacter johnsonii* M19.
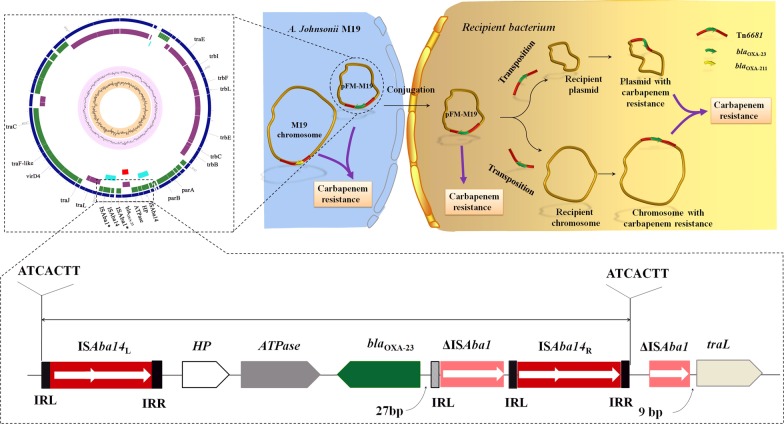

## Introduction

Carbapenems are considered to be reliable and effective antibiotic agents against most pathogenic bacteria because of their broad antibacterial spectrum [1] and are used in the treatment of serious nosocomial infections caused by cephalosporin-resistant bacteria [1]. Species of the *Acinetobacter* genus are extremely well adapted to the hospital environment and can easily become resistant to available antimicrobial agents; therefore, the isolation of carbapenem-resistant *Acinetobacter* species has raised increasing concerns [2–6]. *Acinetobacter johnsonii* is an opportunistic human pathogen that colonizes humans but rarely causes clinical infections. Nevertheless, verification of a carbapenem-resistant strain of *A. johnsonii* encoding an extended-spectrum β-lactamase raises concern [7, 8].

β-lactamases are common mediators of β-lactam resistance and have been divided into four classes: A, B, C and D [2]. Members of class D, which are also referred to oxacillinases (OXAs), are notable contributors to carbapenem resistance and have been frequently observed in *Acinetobacter* species [9]. OXAs with carbapenemase activity were classified into 12 subgroups based on their amino acid sequences [10], and OXA-23 is the major source of carbapenem resistance in *Acinetobacter* [11].

From the bacterial perspective, conjugative plasmids are an ideal vehicle for transferring resistance genes among species. Fortunately, only a few types of plasmids in *Acinetobacter* species are conjugative and able to transfer resistance genes into new hosts [12]. However, numerous transposons, such as Tn*2006*, Tn*2007*, Tn*2008* and Tn*2009*, have frequently been found to be associated with OXA genes [13]. The migration of OXA genes onto transposons has allowed them to become transmissible factors [14]. In this study, we isolated the high-level carbapenem-resistant strain *A. johnsonii* M19 from hospital sewage and discovered that it contained a novel transposon in a conjugative plasmid, thus allowing us to explore the potential for dissemination of carbapenem resistance by this species. These results provide new insights into the mechanisms of dissemination of carbapenem resistance.

## Materials and methods

### Isolation and identification of the carbapenem-resistant strain M19

Hospital sewage was obtained from the influx of the wastewater treatment facility in Shandong province, China. The sewage samples were diluted and spread onto Luria–Bertani (LB) agar plates containing 8 mg/L meropenem (Sigma Co. Shanghai, China) and then incubated at 30 °C for 24 h. A single clone, named M19, was isolated and cultured in LB medium containing meropenem at 30 °C overnight and stored in 15% glycerin at − 20 °C.

A partial fragment of the 16S rRNA gene of M19 was amplified with the universal primers 27F (5′-agagtttgatcctggctcag-3′) and 1492R (5′-ggttaccttgttacgactt-3′) and sequenced. Similarity analyses of the 16S rRNA sequences were conducted using BLASTn (https://blast.ncbi.nlm.nih.gov/Blast.cgi). A phylogenetic tree was produced by using the neighbor-joining algorithms with the Molecular Evolutionary Genetics Analysis 7 (MEGA 7) software based on BLAST results of the 16S rRNA sequence [15]. Antimicrobial susceptibility tests were performed to determine the MICs for carbapenems based on the breakpoints defined by the Clinical and Laboratory Standards Institute [4].

### Whole-genome sequencing, annotation and analysis

The M19 genome was sequenced by PacBio RS II and Illumina HiSeq 4000 platforms at BGI Co., Ltd. (Wuhan, China). Gene prediction was performed on the M19 genome assembly by glimmer3 (https://www.cbcb.umd.edu/software/glimmer/) with Hidden Markov models [16]. Genome annotation was performed using the Prokaryotic Genome Annotation Pipeline on NCBI (https://ncbi.nlm.nih.gov/genome/annotation_prok/). Virulence factors and pathogenicity analysis were identified based on the core dataset in the Virulence Factors of Pathogenic Bacteria database (VFDB) [17] and the Pathogen Host Interactions (PHI) database [18].

### Bioinformatics analyses of resistance genes, transposon and conjugation system

Antibiotic resistance genes (ARGs) were analyzed by RAST and BLASTp based on the core dataset in the Antibiotic resistance genes database (ARDB) [19]. Multi-sequence comparison was carried out by Clustal Omega [20] and ESPript [21]. Homologous model construction was operated by Discovery Studio 2.0 [22]. Molecular docking was performed by the CDOCKER protocol of Discovery Studio 2.0 [22]. IS transposases were detected by IS-Finder [23]. The new transposon was denominated and registered as Tn*6681*, according to the Transposon Registry (https://transposon.lstmed.ac.uk/). The genetic context of Tn*6681* was compared with Tn*2008* and Tn*2008B* using BLASTn. The conjugation system was identified by PATRIC server [24] and oriTfinder [25].

### Mating experiments

Broth-based mating experiments were carried out using M19 as the donor and *Escherichia coli* 25DN as the recipient as described previously [26]. M19 and 25DN were cultivated overnight in LB medium containing 8 mg/L meropenem and 220 mg/L sodium azide. The mixture was incubated at 37 °C for 30 min, and transconjugants were selected on plates containing 8 mg/L meropenem, 220 mg/L sodium azide and 0.1 mg/L 5-bromo-4-chloro-3-indolyl-beta-D-glucuronic acid. The conjugal transfer efficiency was calculated, and six transconjugants, named MAT-1 to MAT-6, were isolated and purified. The MICs of carbapenems for these transconjugants were determined as above. To determine whether the plasmid pFM-M19 and *bla*_OXA-23_ were transferred to transconjugants, DNA fragments of the plasmid in transconjugants MAT-1 to MAT-6 were extracted and used as templates. The transconjugants were analyzed by PCR with a primer pair (Plasmid-For: 5′-tgtataggtgtgatgccttgta-3′; Plasmid-Rev: 5′-agaaacacagtgatgggagata-3′) for pFM-M19 or a primer pair (OXA23-For: 5′-ctgtcaagctcttaaataatattcagc-3′; OXA23-Rev: 5′-tattcgtcgttagaaaaacaattattg-3′) for the *bla*_OXA-23_ gene, and DNA sequencing was performed to confirm the presence of *bla*_OXA-23_ and plasmid-related genes.

## Results

### *Acinetobacter johnsonii* M19 has high carbapenem resistance

**Fig. 1:**
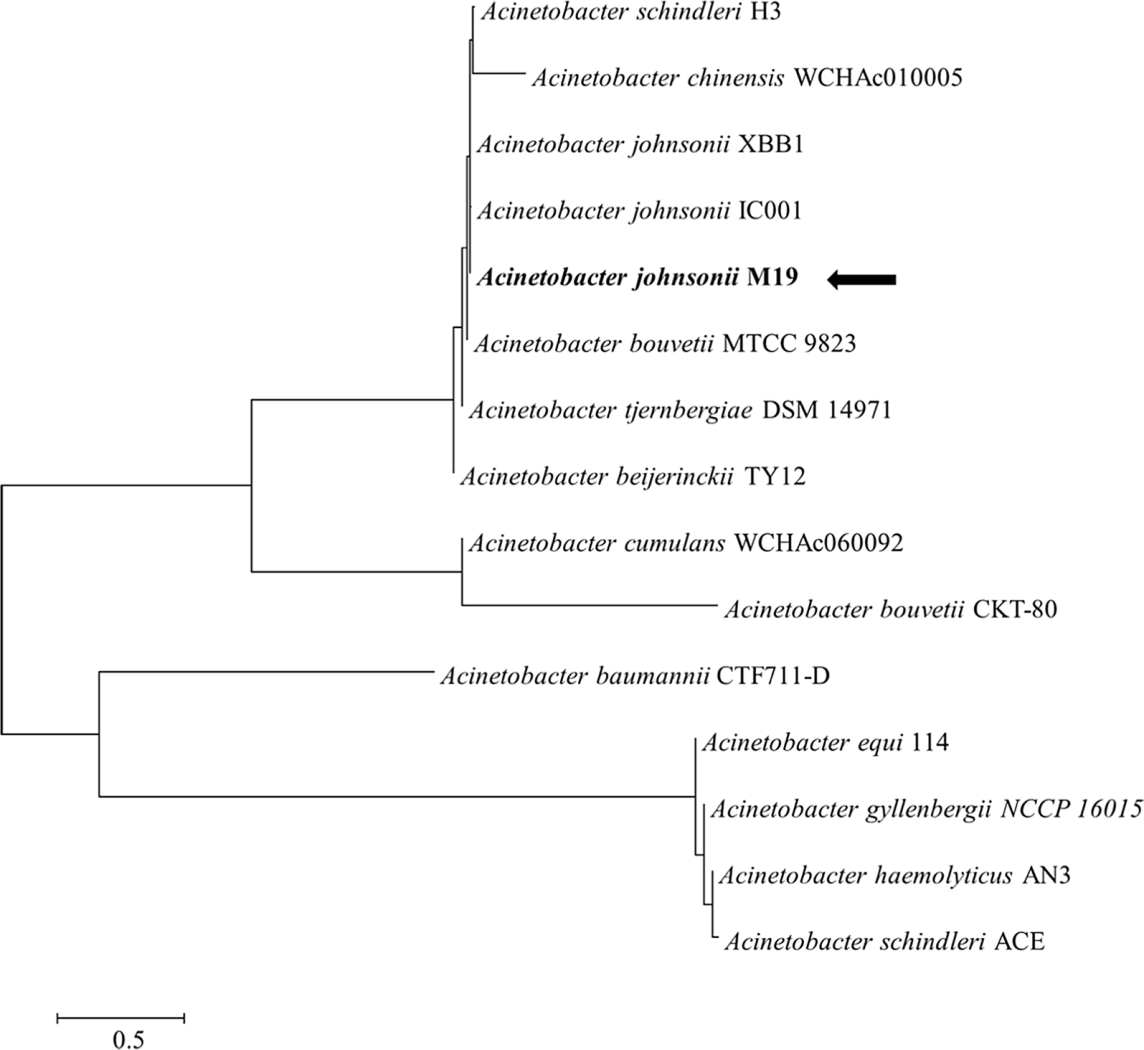
Neighbor-joining tree generated on the basis of 16S rDNA gene sequences and showing the relationship of M19 to other *Acinetobacter* species. Bootstrap values are shown as percentages of 1000 replicates when those values were greater than 50%. The scale bar represents 0.5% substitution per nucleotide position

Strain M19 was isolated from hospital sewage and identified as *A. johnsonii* based on the 16S rDNA sequence (Fig. 1). MICs of imipenem, meropenem and ertapenem for *A. johnsonii* M19 were 128 mg/L, 48 mg/L and 24 mg/L, respectively, which were higher than those reported for most *A. johnsonii* strains (Table 1) [4, 27–32], indicating that strain M19 had striking resistance to carbapenems.

**Table 1:** MICs of carbapenems for the *A. johnsonii* strains

Strains	Carbapenem resistance MIC (mg/L)			References
	IPM	MEM	ERP	
*A. johnsonii* M19	≥ 128	48	24	This work
*A. johnsonii* XBB1	4	≥ 2	/	[27]
*A. johnsonii* XBC1	4	≥ 2	/	[27]
*A. johnsonii* Aj306, Aj289, Aj286, Aj205	≤ 1	≤ 0.25	/	[28]
*A. johnsonii* CIP70.16	0.12	0.19	3	[4]
*A. johnsonii* 2199	2	2	/	[29]
*A. johnsonii* 370, 371, 372, 373	/	≥ 128	/	[30]
*A. johnsonii* 363, 366, 364, 367	/	0.25–1	/	[30]
*A. johnsonii* ATCC 17909	/	2	/	[31]
*A. johnsonii* Z4SZ2	0.125	0.19	/	[32]
*A. johnsonii* ST-2	0.75	0.38	/	[32]
*A. johnsonii* J6	0.5	0.19	/	[32]
*A. johnsonii* 6/1	0.5	0.38	/	[32]

IPM, imipenem; MEM, meropenem; ERP, ertapenem; /, not determined

The whole genome of M19 was sequenced, and the assembled genome contained one 3.75 Mb circular chromosome with 41.4% GC content, and one 55 kb circular plasmid, here named pFM-M19, with 35.8% GC content. The general features of the complete genome sequence are included in Additional file 1: Table S1. Overall, 197 genes (5.24% of the total genes) could be assigned to a VFDB number, and 228 genes (6.07% of the total genes) to a PHI number, indicating that M19 has a high pathogenic potential for humans or other hosts.

Dozens of ARGs were identified in the genome of M19 (Additional file 1: Table S2). Three classes of β-lactamase-encoding genes (class B, class C and class D) were identified, including genes encoding six metallo-β-lactamases (MBLs), two AmpCs and two OXAs (OXA-23 and OXA-211). Furthermore, other antibiotic resistance genes, including efflux pumps, a porin and an aminoglycoside-modifying enzyme gene, were also identified.

### M19 harbours two oxacillinases genes, *bla*_OXA-23_ and *bla*_OXA-211_

Genome annotation of *A. johnsonii* M19 revealed the presence of two OXA-encoding genes, which are responsible for carbapenem resistance, *bla*_OXA-211_ in the chromosome and *bla*_OXA-23_ in plasmid pFM-M19. In addition, *bla*_OXA-211_ in M19 has the same genetic context conserved in other *A. johnsonii* strains (Additional file 1: Fig. S1) and which appears to be ubiquitous in this species [28].

OXA-23 encoded by plasmid pFM-M19 exhibited extremely high amino acid identity with OXA-23 found in *A. baumannii* (CAB69042.1), *A. pittii* (AUF80820.1), *A. wuhouensis* (AYO52469.1), *A. indicus* (ANG65640.1), *A. nosocomialis* (AKL90363.1), *E. coli* 521 (AIE13834.1), *A. baylyi* (AER61544.1), *A. radioresistens* (ABX00637.1) and *Klebsiella pneumoniae* (WP_063864531.1) (Additional file 1: Fig. S2). M19 OXA-23 also has the conserved active-sites (Additional file 1: Fig. S2) (for example Ser-79, Ser-126, Lys-216, Phe-110 and Met-221) that essential to carbapenemase activity [33, 34].

**Fig. 2:**
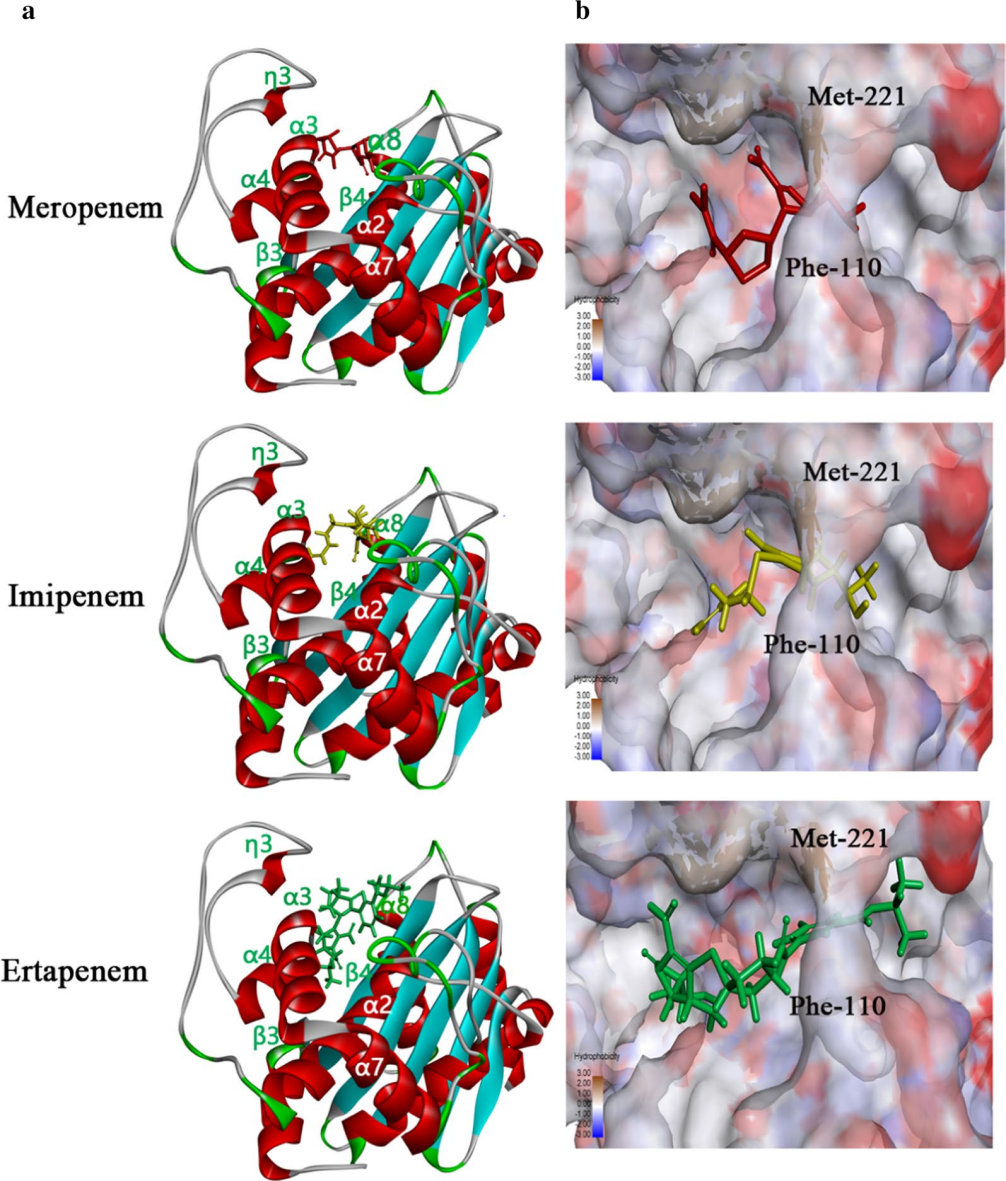
Tertiary structure modelling of *A. johnsonii* M19 OXA-23 with carbapenems. **a** Molecular docking models showing embedding of carbapenems in the OXA-23 cavity. **b** Hydrophobicity of OXA-23 surface and hydrophobic tunnel. Meropenem, imipenem and ertapenem are represented, respectively, by red, blue and green stick models

The tertiary structure of M19 OXA-23 was modelled based on the crystal structure of 4JF4, which is an OXA-23 from *A. baumannii* (GenBank accession number CAB69042.1) and which had the highest amino acid similarity with M19 OXA-23 in the Protein Data Bank (Fig. 2). In this model, the hydrophobic tunnel was formed by Phe-110 and Met-221, and had an elongated shape. Meropenem, imipenem and ertapenem were able to traverse the hydrophobic tunnel and bound to similar positions in the tunnel (Fig. 2). Additionally, Phe-110, Lys-216, Thr-217, Met-221 and Arg-259 were the conserved reactive amino acids (Additional file 1: Fig. S3).

### ***bla***_OXA-23_ is located in the novel transposon Tn***6681*** in pFM-M19

**Fig. 3:**
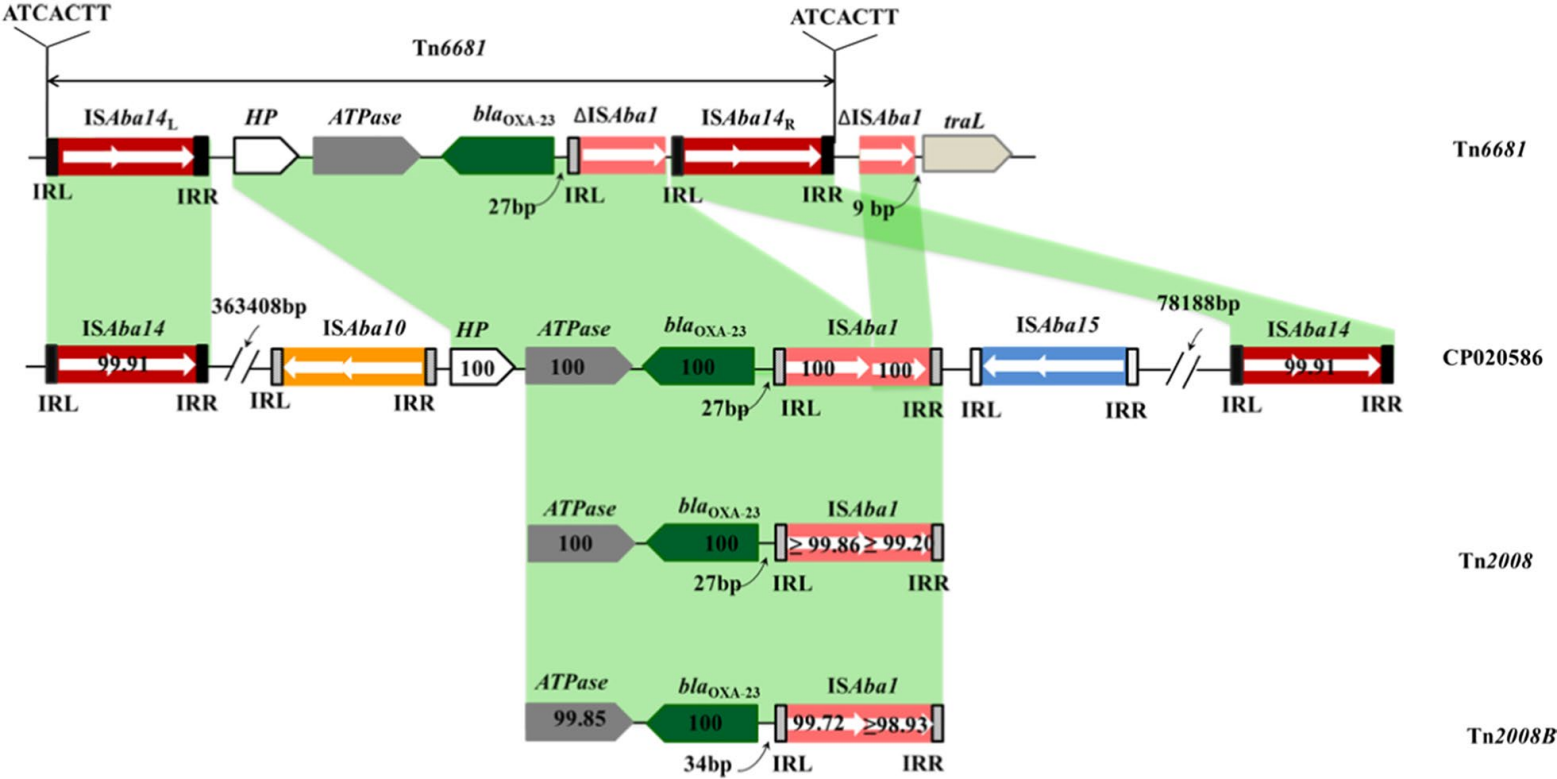
Linear comparison of structures harbouring *bla*_OXA-23_. IRR, right inverted repeat; IRL, left inverted repeat. All block arrows indicate length and orientation. The numbers in each block arrow or box represent the nucleotide sequence identity compared with Tn*6681*. The distance between IS*Aba1* or ΔIS*Aba1* and the *bla*_OXA-23_ start codon is indicated by the number of base pairs below a curved arrow. Direct repeat sequences are indicated

To evaluate the potential for horizontal transfer of *bla*_*OXA-23*_, the genetic context of *bla*_OXA-23_ was investigated. Notably, sequence analysis found that the region containing *bla*_OXA-23_ formed a composite transposon with the components IS*Aba14*-*HP*-*ATPase*-*bla*_OXA-23_-ΔIS*Aba1*-IS*Aba14* (Fig. 3); this novel transposon has been named Tn*6681* in the Transposon Registry and GenBank (Accession number: MN081614). Further alignment analysis showed that Tn*6681* was highly similar to a chromosome fragment of *A. baumannii* CBA7, which was isolated in Korea (Accession number: CP020586.1) [35]. However, the *bla*_OXA-23_ context region of CBA7 is IS*Aba10*-*HP*-*ATPase*-*bla*_OXA-23_-IS*Aba1*-IS*Aba15*, which differs somewhat from Tn*6681*. In addition, two IS*Aba14* genes, marked as IS*Aba14*_L_ and IS*Aba14*_R_, were found upstream (3,63,408 bp) of the IS*Aba10* gene and downstream (78,188 bp) of the IS*Aba15* gene in the CBA7 chromosome and share 99.91% identity with IS*Aba14* in Tn*6681* (Fig. 3). Given their overall similarity, we propose that Tn*6681* and this region of the CBA7 chromosome have the same ancestor.

**Fig. 4:**
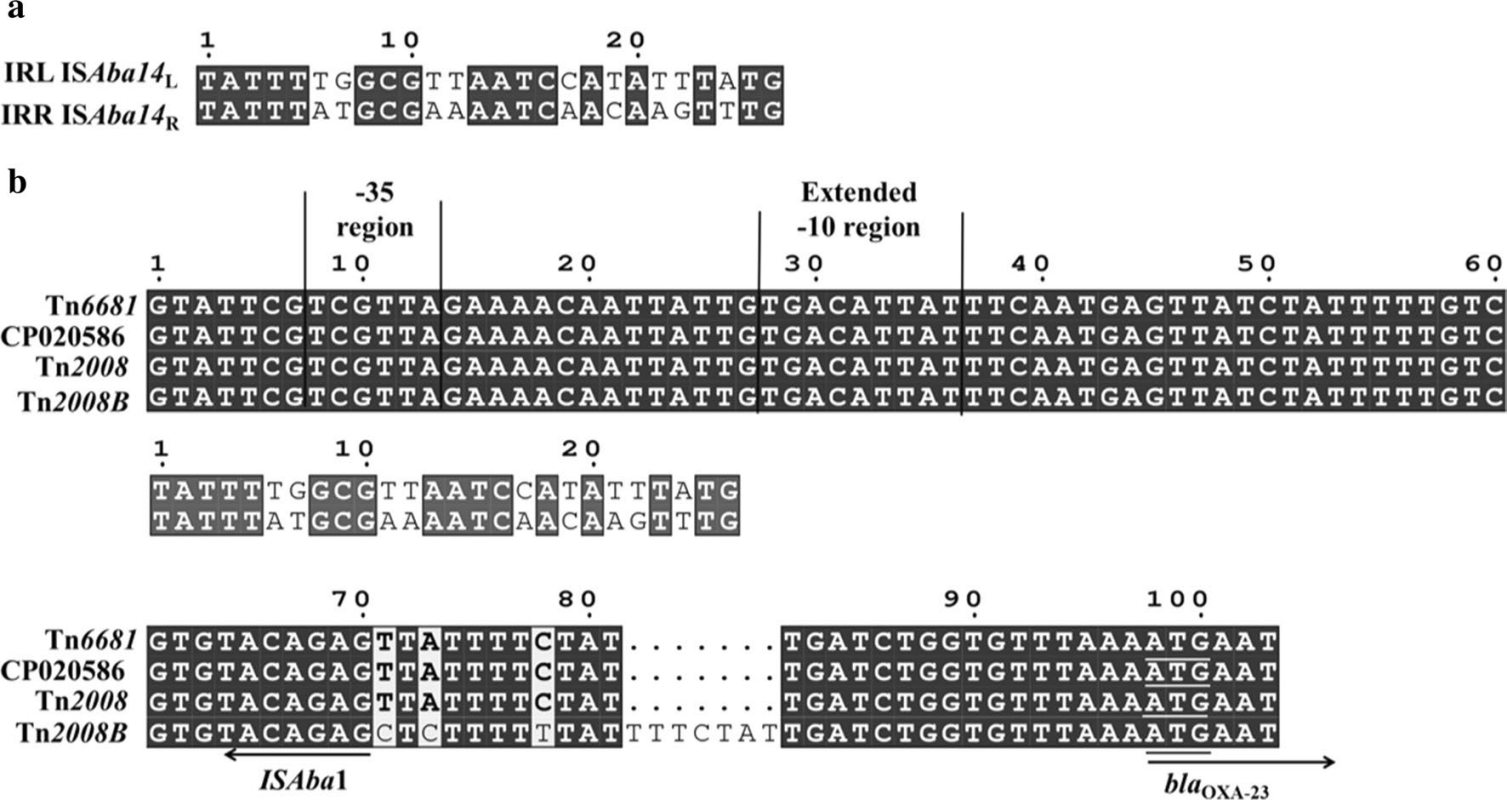
Sequence comparisons of the IS*Aba1* junction upstream of *bla*_OXA-23_. **a** Sequence alignment of left inverted repeat (IRL) of IS*Aba14*_L_ and right inverted repeat (IRR) of IS*Aba14*_R_. **b** Sequence comparisons of the IS*Aba1* junctions upstream of *bla*_OXA-23_ genes. The start codon of the *bla*_OXA-23_ gene is underlined. The − 35 and extended − 10 regions of the promoter are marked

IS*Aba14* genes belong to the IS3 family and have been previously identified as part of the active composite transposon Tn*2114* in *A. baumannii* RAB [36]. Analysis of the inverted repeats (IRs) of IS*Aba1* showed that the right inverted repeat (IRR) of IS*Aba1* remained only 9 bp and the direct repeat and inverted repeat sequences vanished, but the left inverted repeat (IRL) of IS*Aba14*_L_ shared sequence similarity with IRR of IS*Aba14*_R_ (17/26) in particular the motif TATTT(TG/AT)GCG in their extremities (Fig. 4a). The direct repeat sequences (ATCACTT) of 7 bp were also identified (Fig. 3). This overall structure formed a composite transposon Tn*6681*, which is a novel *bla*_OXA-23_ containing transposon.

Additionally, the arrangement *ATPase*-*bla*_OXA-23_-IS*Aba1* constitutes a classic genomic organisation found in Tn*2008* of *A. pittii* (GenBank accession number MF078634) and Tn*2008B* from *A. baumannii* (GenBank accession number LN877214.1) [13]. In Tn*2008*, the promoter of *bla*_OXA-23_ was overlapped by IS*Aba1* upstream of the start codon of OXA-23, and both the -10 and -35 regions of this promoter are within the sequence of the IS*Aba1* gene [37–39]. In Tn*6681*, the insertion of IS*Aba14* into IS*Aba1* generated two ΔIS*Aba1*, but the complete -10 and -35 regions of the *bla*_OXA-23_ promoter were fully maintained (Fig. 4b), indicating that *bla*_OXA-23_ should be expressed normally in M19.

### Conjugative plasmid pFM-M19 disseminates ***bla***_OXA-23_ and carbapenem resistance

**Fig. 5:**
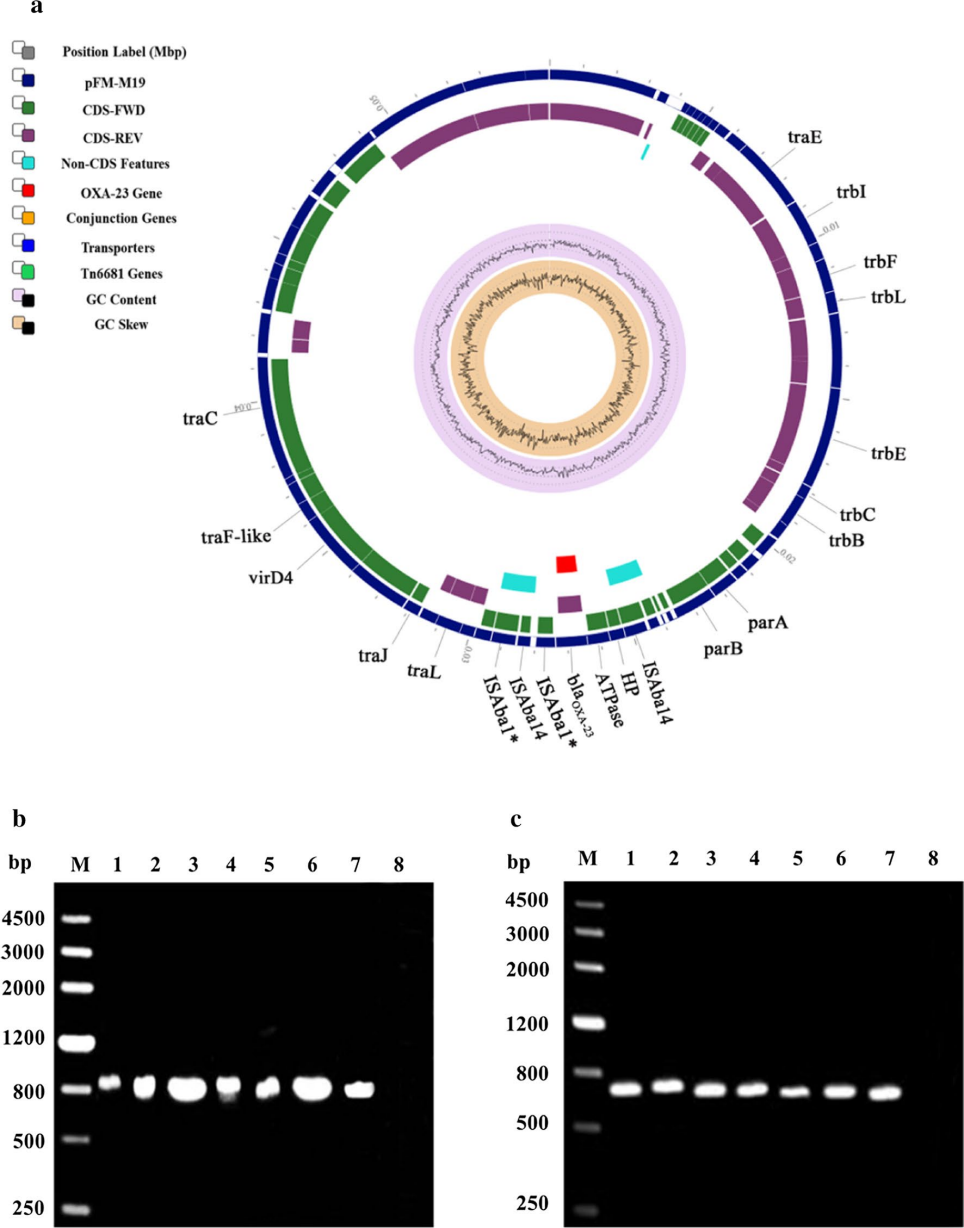
Schematic representation of plasmid pFM-M19 and amplification of *bla*_OXA-23_ and specific region of pFM–M19 from six transconjugant. **a** The asterisk on IS*Aba1* indicates a truncated gene. **b**, **c** M, DNA marker. The following templates were used in PCR: Lanes 1, plasmid fragments extracted from M19; Lanes 2–7, plasmid fragments extracted from the six transconjugants MAT-1 to MAT-6; Lanes 8, genome of 25DN

To evaluate the ability to transfer *bla*_OXA-23_ and carbapenem resistance, the conjugation systems of pFM-M19 were analyzed. Components of conjugative machinery were identified in pFM-M19, such as a relaxase; the type IV coupling protein (T4CP) gene (*traG*) for initiation of conjugation; type IV secretion system (T4SS)-related genes, including the translocation channel protein genes (*trbD*, *trbL*, *trbF*, *trbG* and *trbI*); the pilus protein genes (*trbC* and *trbJ*) and the ATPase genes (*trbE*, *trbB* and *traG*), indicating that pFM-M19 was a conjugative plasmid (Fig. 5a).

Mating experiments between M19 and *E. coli* 25DN were carried out. The results revealed that the conjugal transfer efficiency of pFM-M19 from M19 to *E. coli* 25DN was approximately 1.6 × 10^–4^ CFU/donor when 8 mg/L meropenem was used as the selective pressure. The MICs of carbapenems in six transconjugants were 20 mg/L (imipenem), 16 mg/L (meropenem) and 4 mg/L(ertapenem), which were weaker than that of the donor strain M19 but much higher than that of strain 25DN (Table 2). PCR analysis was performed to confirm the dissemination of carbapenem resistance via plasmid pFM-M19 and *bla*_OXA-23_. The results showed that both pFM-M19 and *bla*_OXA-23_ were detected in all transconjugants (Fig. 5b and c) and suggested that *E. coli* 25DN obtained carbapenem resistance due to the acquisition of *bla*_OXA-23_ along with pFM-M19.

**Table 2:** MICs of carbapenem-resistant strains

Strains	MICs (mg/L)		
	IPM	MEM	ERP
M19	≥ 128	48	24
25DN	< 2	< 2	< 2
MAT-1	20	16	4
MAT-2	20	16	4
MAT-3	20	16	4
MAT-4	20	16	4
MAT-5	20	16	4
MAT-6	20	16	4

25DN, sodium azide-resistant *E. coli* strain derived from ATCC25922; MAT, transconjugants of *A. johnsonii* M19 and *E. coli* 25DN; IPM, imipenem; MEM, meropenem; ERP, ertapenem

## Discussion

*Acinetobacter johnsonii* strain M19 presented higher carbapenem resistance than that of most *A. johnsonii* strains [2, 4, 28–31, 40], *Pseudomonas aeruginosa* [41], *Proteus mirabilis* [42] or *A. baumannii* [43]. β-lactam antibiotic resistance of *Acinetobacter* is mainly due to the inactivation of β-lactams catalysed by four classes (A, B, C and D) of β-lactamases [9, 13]. In this work, three classes (class B, C and D) of β-lactamase genes, including six MBL-encoding genes, two AmpC-encoding genes and two OXA-encoding genes, were identified in the genome of *A. johnsonii* M19, suggesting that M19 is a reservoir of β-lactam resistance genes.

Class D β-lactamases, commonly referred to as OXA, are responsible for carbapenem resistance, and their encoding genes are conserved and widespread in *Acinetobacter* species [44, 45]. M19 harbours *bla*_OXA-211_ in the chromosome and *bla*_OXA-23_ in plasmid pFM-M19. OXA-23 was the first reported class D β-lactamase and was originally detected in a patient isolate of *A. baumannii* in Scotland in 1993 [46]. Twenty years later, the structure of OXA-23 was resolved and revealed that the elongated pocket of OXA-23 provides a hydrophobic environment for high reaction efficiency with carbapenems [11]. OXA-23 is considered to be the major β-lactamase for carbapenem resistance in *Acinetobacter* [11], suggesting that the OXA-23 encoded by plasmid pFM-M19 plays a key role in carbapenem resistance in M19. Interestingly, the *bla*_OXA-23_ found in plasmid pFM-M19 has not been previously reported in *A. johnsonii* strains, indicating that M19 obtained this gene from other bacterial species and further increasing concern over the ability of *bla*_OXA-23_ to spread among species*.*

Mobile elements are considered to be responsible for the movement and dissemination of *bla*_OXA-23_, and all of the reported genetic structures that contain *bla*_OXA-23_ have been classified as transposons [13]. Previously, *bla*_OXA-23_ had been found in five transposons, with IS*Aba1* upstream of the start codon of *bla*_OXA-23_ in four of these (Tn*2006* [47], Tn*2008* [48], Tn*2008B* [49], Tn*2009* [50]) and with IS*Aba4* preceding *bla*_OXA-23_ in Tn*2007 *[47]*.* In plasmid pFM-M19, we found that *bla*_OXA-23_ was located in the new transposon Tn*6681*, which has the genetic context IS*Aba14*-*HP*-*ATPase*-*bla*_OXA-23_-ΔIS*Aba1*-IS*Aba14*-ΔIS*Aba1* and which was likely formed as two copies of IS*Aba14* were inserted into the IS*Aba1* of Tn*2008.* The structure of Tn*6681* has some differences from that of the other transposons previously reported to contain *bla*_OXA-23_ [13]. In addition, in Tn*2008*, a high level of expression of the *bla*_OXA-23_ gene is associated with significant resistance to carbapenems in *A. baumannii*, and this expression is controlled by promoter elements within IS*Aba1* [37–39]*.* However, although sequence analysis revealed that IS*Aba14* inserted into IS*Aba1* in Tn*6681,* the promoter of *bla*_OXA-23_ appears to be intact, indicating that the *bla*_OXA-23_ gene may still be highly expressed and responsible for the striking carbapenem resistance in M19.

**Fig. 6:**
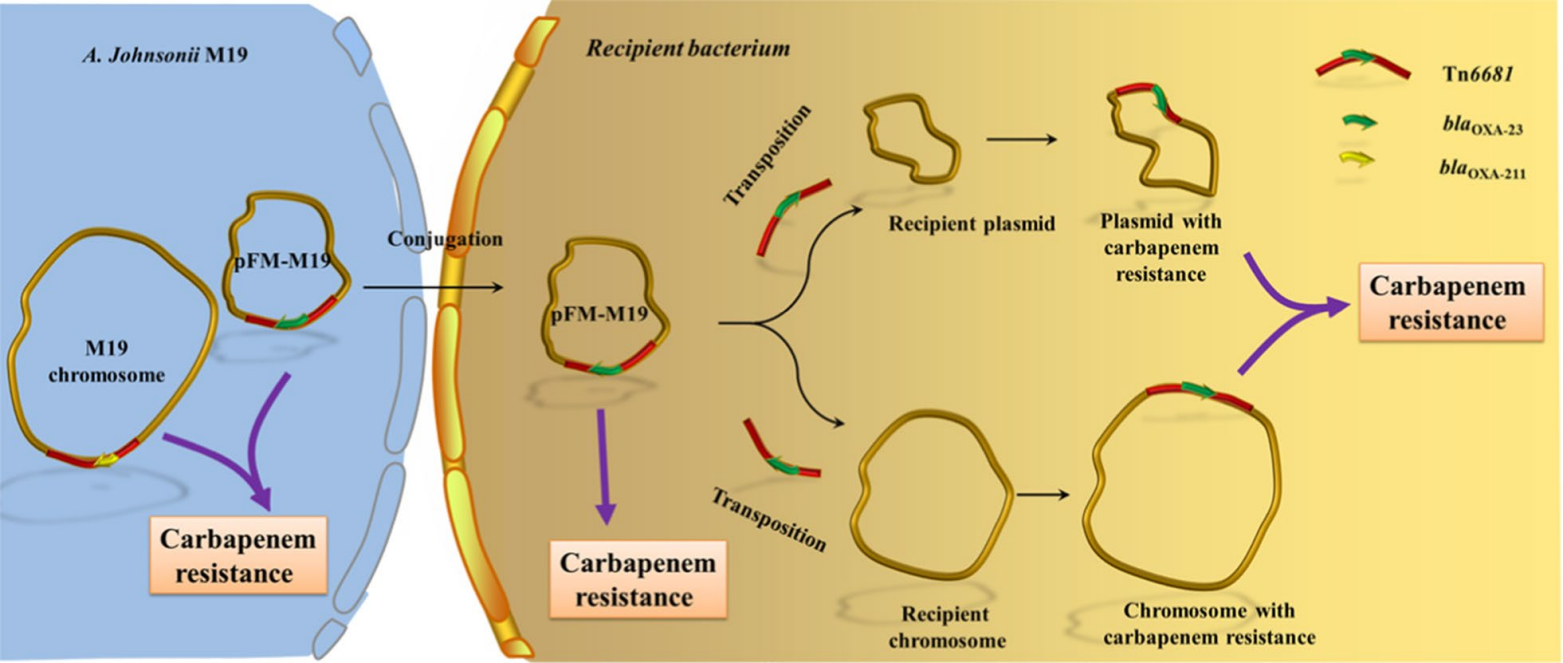
Proposed pathway for transfer of the *bla*_OXA-23_ gene and dissemination of carbapenem resistance from *A. johnsonii* M19

The acquisition of novel genes by plasmids, especially conjugative plasmids [51], along with mobile genetic elements such as transposons or insertion sequences, makes them perfect vehicles for the spread of antibiotic resistance [52]. The conjugative DNA transfer mechanism is well conserved and depends on a T4SS [53, 54]. Genes of T4SS and T4CP modules, including genes for a translocation channel protein, pilus protein and ATPase, were identified in pFM-M19, indicating that pFM-M19 is a conjugative plasmid, which has also been confirmed by mating experiments in this study. However, it should be pointed out that the typical origin of transfer site (*oriT*) was not detected on pFM-M19 by *oriT* finder, suggesting that pFM-M19 might initiate the transfer from a cryptic *oriT*. Moreover, the combination of transposon Tn*6681* and the conjugative plasmid pFM-M19 may provide a robust means for *bla*_OXA-23_ transfer, with the potential for Tn*6681* to shift *bla*_OXA-23_ between the chromosome and plasmid in one bacterial strain, and the conjugative plasmid pFM-M19 disseminating Tn*6681* and *bla*_OXA-23_ between different bacterial species (Fig. 6).

## Conclusions

In conclusion, *A. johnsonii* strain M19, which contains the conjugative plasmid pFM-M19 and Tn*6681*, is not only a reservoir of *bla*_OXA-23_ but also an effective disseminator of *bla*_OXA-23_. To our knowledge, our investigation is the first to provide evidence that *bla*_OXA-23_ was transferred into *A. johnsonii*. The presence of *bla*_OXA-23_ on conjugative plasmids of *A. johnsonii* enhances the risk of carbapenem resistance spread to the environment and needs to be monitored closely.

## Supplementary information

**Additional file 1.** **Table S1.** General features of the *A. johnsonnii* M19 genome. **Table S2.** Antibiotic resistant genes of the *A. johnsonnii* M19 genome. **Table S3.** Predicted genes of plasmid pFM-M19. **Fig. S1.** Comparison of the genetic context of *bla*_OXA-23_ in M19 and other *A. johnsonii* strains. **Fig. S2.** Multi-sequence comparison of OXA-23 proteins from *A. johnsonii* M19 and various other bacteria. **Fig. S3.** Stick models of the active sites of OXA-23 during carbapenem binding and the carbapenem β-lactam ring.

## Data Availability

All data generated or analyzed during this study are included in this published article [and its supplementary information files] Original data are available from the corresponding author upon reasonable request.

## Abbreviations

MICs: Minimum inhibitory concentrations
PATRIC: Pathosystems Resource Integration Center
OXA: Oxacillinase
LB: Luria–Bertani
MEGA: Molecular evolutionary genetics analysis
VFDB: Virulence factors of pathogenic bacteria database
PHI: Pathogen host interactions
ARGs: Antibiotic resistance genes
ARDB: Antibiotic resistance genes database
MBLs: Metallo-β-lactamases
IRs: Inverted repeats
IRR: Right inverted repeat
IRL: Left inverted repeat
T4CP: Type IV coupling protein
T4SS: Type IV secretion system
*oriT*: Origin of transfer site
