# Supplementary material for: The carbapenem resistance gene blaOXA-23 is disseminated by a conjugative plasmid containing the novel transposon Tn6681 in Acinetobacter johnsonii M19
Source: Antimicrob Resist Infect Control. 2020 Nov 9;9:182. doi: 10.1186/s13756-020-00832-4 (PMC7653874; doi:10.1186/s13756-020-00832-4)
Supplement: Supplementary file 1 — Additional file 1. Table S1. General features of the A. johnsonnii M19 genome. Table S2. Antibiotic resistant genes of the A. johnsonnii M19 genome. Table S3. Predicted genes of plasmid pFM-M19. Fig. S1. Comparison of the genetic context of blaOXA-23 in M19 and other A. johnsonii strains. Fig. S2. Multi-sequence comparison of OXA-23 proteins from A. johnsonii M19 and various other bacteria. Fig. S3. Stick models of the active sites of OXA-23 during carbapenem binding and the carbapenem β-lactam ring. [file 13756_2020_832_MOESM1_ESM.docx]

**Supplemental files**

**The carbapenem resistance gene *bla*_OXA-23_ is disseminated by a conjugative plasmid containing the novel** **transposon Tn*6681* in *Acinetobacter johnsonii* M19**

Gongli Zong^1,2,3^, Chuanqing Zhong^4^, Jiafang Fu^2,3,5^, Yu Zhang^4^, Peipei Zhang^2,3,5^, Wenchi Zhang^6^, Yan Xu^1^, Guangxiao Cao^2,3*^, Rongzhen Zhang^1^^[[1]](#footnote-1)^

**Table S1** General features of the *A. johnsonii* M19 genome

| Features | Genome | |
| --- | --- | --- |
|  | Chromosome | plasmid |
| Total number of base pairs | 3,749,210 | 55,044 |
| G+C content (%) | 41.39 | 35.77 |
| Total genes | 3,774 | |
| Number of ncRNA genes | 121 | |
| Number of tandem repeats | 147 | |
| Number of COG annotation genes | 2,613 | |
| Number of GO annotation genes | 2,124 | |
| Number of KEGG annotation genes | 2,030 | |
| Number of VFDB annotation genes | 197 | |
| Number of PHI annotation genes | 228 | |
| Number of ARDB annotation genes | 21 | |

Abbreviations: ncRNA, noncoding RNA; COG, Clusters of Orthologous Groups database; GO, Gene Ontology database; KEGG, Kyoto Encyclopedia of Genes and Genomes; VFDB, Virulence Factors of Pathogenic Bacteria database; PHI, Pathogen Host Interactions database; ARDB, Antibiotic Resistance Genes database.

**Table S2** Antibiotic resistance genes of the *A. johnsonii* M19 genome

| Resistance mechanism | Class/subgroup | Protein | Number of genes | Location |
| --- | --- | --- | --- | --- |
| β-lactamase | Class B | MBL | 6 | Chromosome |
|  | Class C | AmpC | 2 | Chromosome |
|  | **Class D** | **OXA-211** | **1** | **Chromosome** |
|  |  | **OXA-23** | **1** | **Plasmid** |
| Efflux pumps | MFS | MdtD | 1 | Chromosome |
|  |  | Bcr/CflA | 1 | Chromosome |
|  |  | MdfA | 1 | Chromosome |
|  |  | FsR | 1 | Chromosome |
|  | RND | MexP | 1 | Chromosome |
|  |  | MexQ | 1 | Chromosome |
|  |  | OpmE | 1 | Chromosome |
|  |  | AdeA | 1 | Chromosome |
|  |  | AdeB | 1 | Chromosome |
|  |  | AdeC | 1 | Chromosome |
|  |  | AdeI | 1 | Chromosome |
|  |  | AdeJ | 1 | Chromosome |
|  |  | AdeK | 1 | Chromosome |
|  |  | AdeT | 2 | Chromosome |
|  | SMR | QacE | 1 | Chromosome |
|  | MATE | MdtK/NorM | 2 | Chromosome |
|  | PACE | AceI | 1 | Chromosome |
|  | Other efflux pumps | EmrA | 2 | Chromosome |
|  |  | EmrB | 2 | Chromosome |
|  |  | MacA | 1 | Chromosome |
|  |  | MacB | 1 | Chromosome |
|  |  | MacC | 1 | Chromosome |
| Permeability defects | Porin | OmpA | 1 | Chromosome |
| Aminoglycoside-modifying enzymes | Aminoglycoside phosphotransferase | APH(3") | 1 | Chromosome |
| Fluoroquinolones | *gyrA* mutation | GyrA | 1 | Chromosome |
|  | *parC* mutation | ParC | 1 | Chromosome |

MFS, major facilitator superfamily; FsR, Fosmidomycin resistance protein; RND, resistance nodulation division; SMR, small multidrug resistance; MATE, multidrug and toxic compound extrusion; PACE, proteobacterial antimicrobial compound efflux.

**Table S3** Predicted genes of plasmid pFM-M19

| Gene ID | Start | Stop | Strand | Protein name |
| --- | --- | --- | --- | --- |
| E0Z08_18620 | 11 | 3286 | - | ATP-dependent helicase |
| E0Z08_18630 | 5442 | 5879 | - | hypothetical protein |
| E0Z08_18635 | 6047 | 6439 | - | hypothetical protein |
| E0Z08_18640 | 6432 | 8603 | - | DNA topoisomerase III |
| E0Z08_18645* | 8683 | 10140 | - | P-type conjugative transfer protein TrbI |
| E0Z08_18650 | 10141 | 10584 | - | hypothetical protein |
| E0Z08_18655* | 10597 | 11628 | - | P-type conjugative transfer protein TrbG |
| E0Z08_18660* | 11656 | 12360 | - | conjugal transfer protein TrbF |
| E0Z08_18665* | 12427 | 13809 | - | P-type conjugative transfer protein TrbL |
| E0Z08_18670* | 13870 | 14646 | - | conjugal transfer protein TrbJ |
| E0Z08_18675* | 14687 | 15085 | - | hypothetical protein |
| E0Z08_18680* | 15057 | 17546 | - | ATPase TrbE |
| E0Z08_18685* | 17559 | 17846 | - | translocation channel protein TrbD |
| E0Z08_18690* | 17900 | 18277 | - | conjugal transfer protein TrbC |
| E0Z08_18695* | 18288 | 19238 | - | P-type conjugative transfer ATPase TrbB |
| E0Z08_18700 | 19846 | 20340 | + | hypothetical protein |
| E0Z08_18705 | 20560 | 21015 | + | hypothetical protein |
| E0Z08_18710 | 21067 | 21402 | + | hypothetical protein |
| E0Z08_18715 | 21476 | 22246 | + | ParA family protein |
| E0Z08_18720 | 22264 | 23523 | + | ParB/RepB/Spo0J family partition protein |
| E0Z08_18725 | 23591 | 23836 | + | hypothetical protein |
| E0Z08_18730** | 24105 | 25249 | + | IS3-like element ISAba14 family transposase |
| E0Z08_18735** | 25312 | 25659 | + | hypothetical protein |
| E0Z08_18740** | 25677 | 26336 | + | ATP-binding protein |
| **E0Z08_18745**** | **26441** | **27262** | **-** | **carbapenem-hydrolysing class D beta-lactamase OXA-23** |
| E0Z08_18750** | 27361 | 28079 | + | IS4 family transposase IS*Aba1* |
| E0Z08_18755** | 28161 | 29305 | + | IS3-like element IS*Aba14* family transposase |
| E0Z08_18760 | 29362 | 29736 | + | hypothetical protein |
| E0Z08_18765 | 29797 | 30282 | - | hypothetical protein |
| E0Z08_18770* | 30292 | 31017 | - | conjugal transfer protein TraL |
| E0Z08_18775 | 31033 | 31395 | - | hypothetical protein |
| E0Z08_18780 | 31723 | 32109 | + | LysR family transcriptional regulator |
| E0Z08_18785* | 32119 | 34026 | + | relaxase |
| E0Z08_18790* | 34113 | 36038 | + | conjugal transfer protein TraG/ VirD4 |
| E0Z08_18795 | 36038 | 36625 | + | signal peptidase I |
| E0Z08_18800 | 36658 | 37302 | + | lytic transglycosylase domain-containing protein |
| E0Z08_18805 | 37316 | 37504 | + | hypothetical protein |
| E0Z08_18810 | 37507 | 41289 | + | DUF1738 domain-containing protein |
| E0Z08_18815 | 41514 | 41966 | - | hypothetical protein |
| E0Z08_18820 | 41994 | 42647 | - | recombinase family protein |
| E0Z08_18825 | 42831 | 43775 | + | hypothetical protein |
| E0Z08_18830 | 43778 | 44257 | + | hypothetical protein |
| E0Z08_18835 | 44270 | 44542 | + | hypothetical protein |
| E0Z08_18840 | 44557 | 45537 | + | hypothetical protein |
| E0Z08_18850 | 46721 | 47497 | + | SprT domain-containing protein |
| E0Z08_18855 | 47799 | 49145 | + | hypothetical protein |
| E0Z08_18860 | 49267 | 52470 | - | DEAD/DEAH box helicase |
| E0Z08_18865 | 52483 | 54357 | - | site-specific DNA-methyltransferase |
| E0Z08_18870 | 54374 | 55018 | - | DUF4391 domain-containing protein |

*, conjugal transfer protein genes; **, genes of Tn*6681*


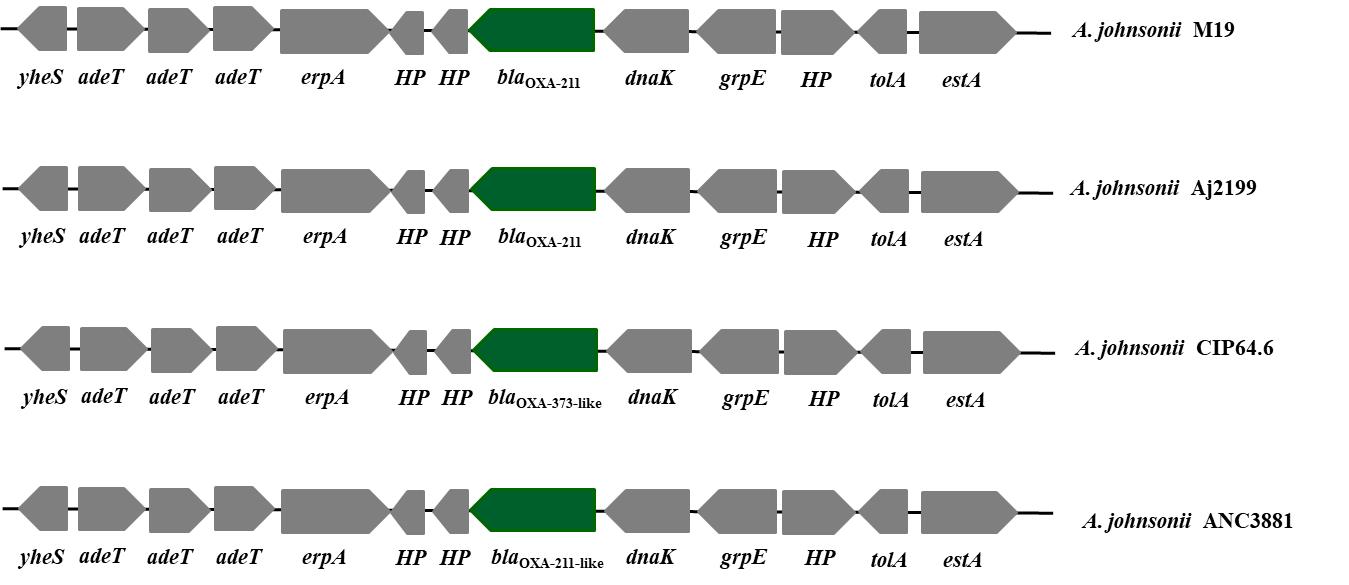


**Fig. S1** Comparison of the genetic context of *bla*_OXA-211_ in M19 and other *A. johnsonii* strains. *bla*_OXA-211_ gene, green arrows; downstream and upstream genes, grey arrows. HP, hypothetical protein.

**
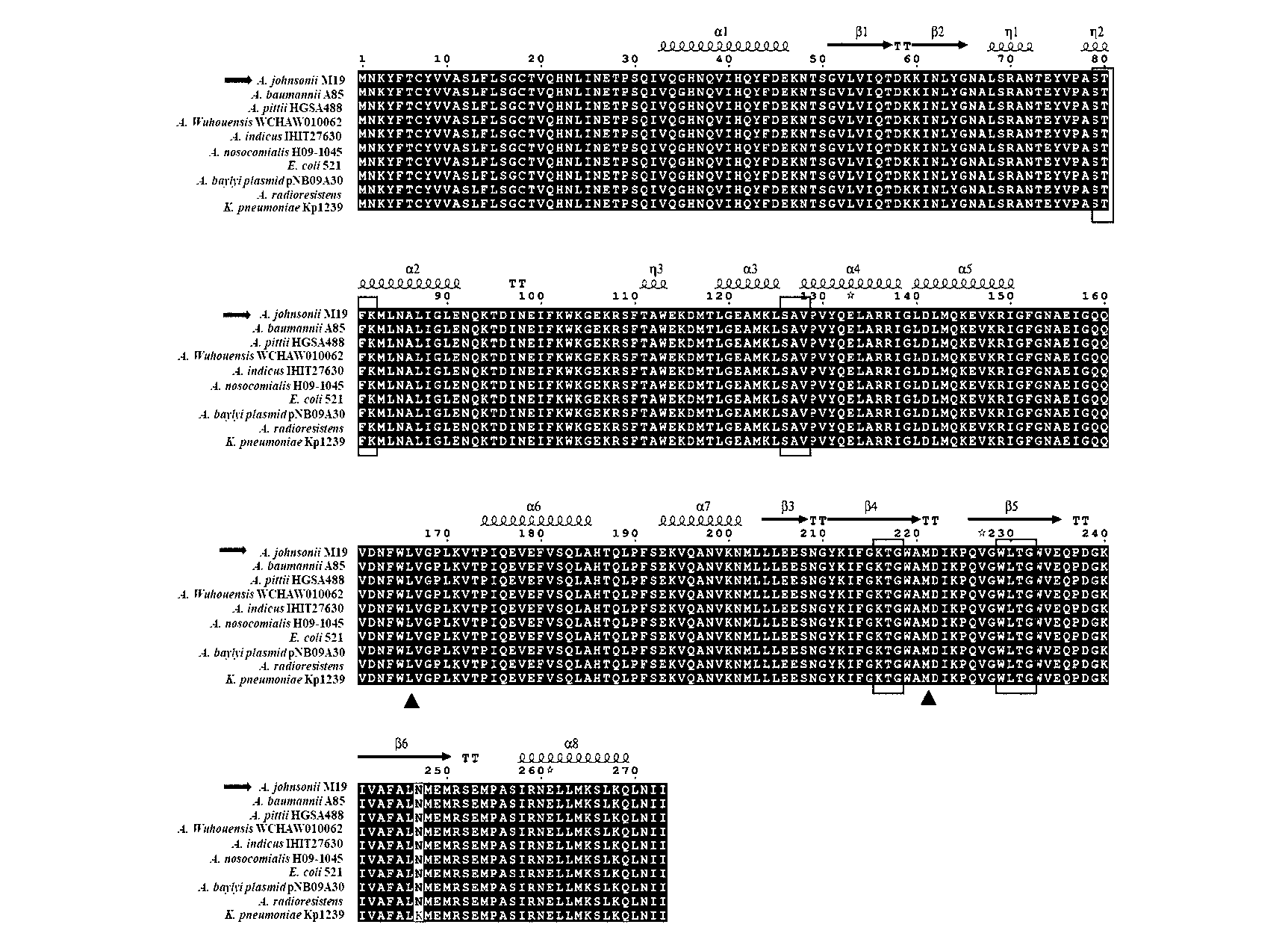
**

**Fig. S2** Multi-sequence comparison of OXA-23 proteins from *A. johnsonii* M19 and various other bacteria. *A. johnsonii* M19, QBK71541.1; *A. baumannii* A85*,* CAB69042.1; *A. pittii* HGSA488, AUF80820.1; *A. wuhouensis* WCHAW010062, AYO52469.1; *A. indicus* IHIT27630, ANG65640.1; *A. nosocomialis* H09-1045, AKL90363.1; *E. coli* 521, AIE13834.1; *A. baylyi* plasmid pNB09A30, AER61544.1; *A. radioresistens*, *ABX00637.1*; *K. pneumoniae* Kp1239, WP_063864531.1. Residues in boxes are known to play a role in the catalytic mechanism, and residues marked by arrowheads are critical for carbapenemase activity. Protein structural features are indicated at the top of each section.


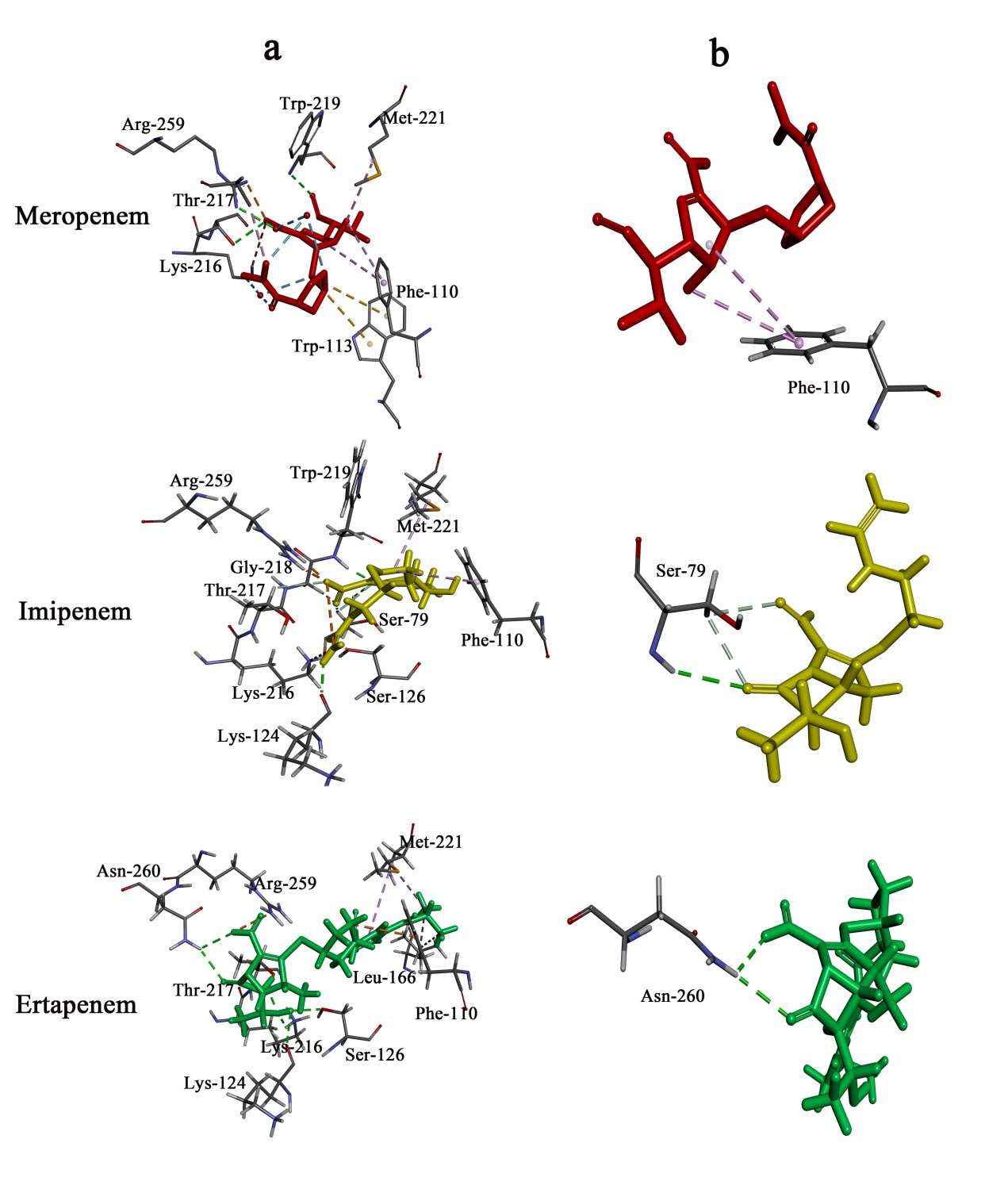


**Fig. S3** Stick models of the active sites of OXA-23 during carbapenem binding and the carbapenem β-lactam ring. Meropenem, imipenem and ertapenem are represented, respectively, by red, blue and green stick models. Broken lines represent bonds between OXA-23 and the carbapenems.

1. Correspondence:

   [rzzhang@jiangnan.edu.cn](mailto:rzzhang@jiangnan.edu.cn) (Rongzhen Zhang);

   ^1^Key Laboratory of Industrial Biotechnology of Ministry of Education & School of Biotechnology, Jiangnan University, Wuxi 214122, China.

   [caozhong0402@163.com](mailto:caozhong0402@163.com) (Guangxiao Cao)

   ^2^ Department of Epidemiology, the First Affiliated Hospital of Shandong First Medical University, Jinan 250062, China. ^3^Shandong Medicinal Biotechnology Center, Shandong First Medical University & Shandong Academy of Medical Sciences, Jinan 250062, China. [↑](#footnote-ref-1)
